# White matter changes should not exclude patients with idiopathic normal pressure hydrocephalus from shunt surgery

**DOI:** 10.1186/s12987-022-00338-8

**Published:** 2022-05-23

**Authors:** Carl Snöbohm, Filip Malmberg, Eva Freyhult, Kim Kultima, David Fällmar, Johan Virhammar

**Affiliations:** 1 Department of Medical Sciences, Neurology, Uppsala University, Uppsala, Sweden; 2 Department of Information Technology, Division of Visual Information and Interaction, Uppsala University, Uppsala, Sweden; 3 Department of Cell and Molecular Biology, Uppsala University, Uppsala, Sweden; 4 Department of Medical Sciences, Clinical Chemistry, Uppsala University, Uppsala, Sweden; 5 Department of Surgical Sciences, Neuroradiology, Uppsala University, Uppsala, Sweden

**Keywords:** Idiopathic normal pressure hydrocephalus, Magnetic resonance imaging, Volumetric segmentation, White matter changes, Shunt surgery outcome

## Abstract

**Introduction:**

White matter changes (WMC) on brain imaging can be classified as deep white matter hyperintensities (DWMH) or periventricular hyperintensities (PVH) and are frequently seen in patients with idiopathic normal pressure hydrocephalus (iNPH). Contradictory results have been reported on whether preoperative WMC are associated with outcome after shunt surgery in iNPH patients. The aim of this study was to investigate any association between DWMH and PVH and shunt outcome in patients with iNPH, using magnetic resonance volumetry.

**Methods:**

A total of 253 iNPH patients operated with shunt surgery and clinically assessed before and 12 months after surgery were included. All patients were investigated preoperatively with magnetic resonance imaging of the brain. The volumes of DWMH and PVH were quantified on fluid-attenuated inversion recovery images using an in-house semi-automatic volumetric segmentation software (SmartPaint). Shunt outcome was defined as the difference in symptom score between post- and preoperative investigations, measured on the iNPH scale, and shunt response was defined as improvement with ≥ 5 points.

**Results:**

One year after shunt surgery, 51% of the patients were improved on the iNPH scale. When defining improvement as ≥ 5 points on the iNPH scale, there was no significant difference in preoperative volume of WMC between shunt responders and non-responders. If outcome was determined by a continuous variable, a larger volume of PVH was negatively associated with postoperative change in the total iNPH scale (p < 0.05) and negatively associated with improvement in gait (p < 0.01) after adjusting for age, sex, waiting time for surgery, preoperative level of symptoms, Evans’ index, and disproportionately enlarged subarachnoid space hydrocephalus. The volume of DWMH was not associated with shunt outcome.

**Conclusions:**

An association between outcome after shunt surgery and volume of PVH was seen, but there was no difference between shunt responders and non-responders in the volumes of DWMH and PVH. We conclude that preoperative assessment of WMC should not be used to exclude patients with iNPH from shunt surgery.

## Introduction

Idiopathic normal pressure hydrocephalus (iNPH) is a neurological disease of the elderly, causing enlargement of the brain ventricles, without radiological signs of cerebrospinal fluid (CSF) obstruction or altered intracranial pressure. The main symptoms of iNPH are gait disturbance, cognitive deficits, and urinary dysfunction [1, 2]. The pathophysiology of iNPH remains controversial, but changes in CSF dynamics such as increased resistance to CSF outflow and increased intracranial pressure pulse amplitudes, combined with cerebrovascular changes, are frequently reported [3–5]. Approximately 60–80% of patients clinically improve after shunt surgery [6–8].

Findings of white matter changes (WMC) on brain imaging are more frequent in iNPH patients compared with healthy age-matched controls [9–11]. On magnetic resonance imaging (MRI), WMC can be visually classified as deep white matter hyperintensities (DWMH) or periventricular hyperintensities (PVH), but the classes are sometimes confluent and impossible to separate on conventional imaging. Several definitions for distinguishing DWMH from PVH have been proposed (for an overview, see Kim et al. [12]), but a widely accepted strategy is to use the continuity-to-ventricle rule as a definition for PVH [13]. DWMH and PVH have differing histopathological findings and dissimilar etiologies and relations to other diseases [12]. Briefly, DWMH are believed to be primarily caused by ischemia secondary to arteriosclerotic small vessel disease [14], while irregular PVH have been associated with large vessel disease, such as carotid atherosclerosis [13], and smooth PVH (caps and halo phenomena) with subependymal gliosis and discontinuity of the ependymal lining [14]. Vascular risk factors are overrepresented in iNPH patients [4, 5, 15, 16].

Beyond clinical examination, various invasive procedures and radiological markers, with limited prognostic values, are typically used to predict shunt responsiveness in iNPH patients [17–19]. Given the discomfort to patients from invasive testing and the risks associated with shunt surgery [6, 7, 20–23], more noninvasive prognostic tools are desirable. Inconsistent results have been published regarding the prognostic value of WMC on shunt outcome. It has been reported that WMC lack prognostic importance [9, 24], but there are also studies suggesting that WMC are associated with either favorable outcomes after shunt surgery [25–27] or unfavorable ones [28, 29]. However, the majority of these studies have used non-volumetric, subjective rating scales for grading the extent of WMC [9, 24, 25, 29].

The aim of this study was to investigate the separate predictive values of preoperative DWMH and PVH on outcome after shunt surgery in a large series of iNPH patients using magnetic resonance volumetry. Volumes were quantified using SmartPaint, an in-house semi-automatic volumetric segmentation software [30].

## Methods

### Study design and population

This retrospective observational single-center study included 262 patients operated with shunt surgery for iNPH between 2011 and 2015. Inclusion criteria were: diagnosis of iNPH based on the international guidelines [31], clinical evaluations before and 12 months after shunt surgery, and a preoperative MRI of the brain including a fluid-attenuated inversion recovery (FLAIR) sequence. The median time between the preoperative MRI and surgery was 10 months (interquartile range (IQR) 7–14) and between the preoperative clinical evaluation and surgery 6 months (IQR 4–8).

Fifty-one (20%) of the included patients suffered from at least one shunt related complication during the first postoperative year: 20 (8%) subdural hematomas/hygromas, 27 (11%) displaced or migrated shunt catheters (13 proximal and 14 distal), 2 (1%) intracerebral hematomas, and 5 (2%) shunt infections. If a shunt failure was suspected, a new postoperative visit was planned and the latest postoperative visit was included in statistical analysis.

Six patients who suffered from non-shunt-related events were excluded from the present study due to the possible impact on pre- and postoperative clinical evaluations. One patient had a fall accident before the preoperative evaluation, two patients sustained hip fractures between the preoperative evaluation and shunt surgery, one patient sustained a hip fracture after surgery, one patient had a stroke between the preoperative evaluation and surgery, and one patient contracted viral encephalitis between the preoperative evaluation and surgery. Three patients were excluded due to radiological artifacts impairing image analysis. Thus, a total of 253 patients, 141 males and 112 females with a median age of 75 years (range 50–89), were included in the statistical analyses. Since this was a retrospective study, patients from the investigated cohort have also been included in past studies at the same center [11, 23, 32, 33]. The Swedish Ethical Review Authority approved the study (Dnr 2015/174/3 and 2019-06566).

### Clinical assessments

All patients were evaluated using at least one domain of the iNPH scale [34] at baseline and 12 months after surgery, and most patients (*n* = 241) were also evaluated with the Mini-Mental State Examination (MMSE). The Swedish iNPH scale consists of four domains based on the most common symptoms of iNPH: gait, balance, neuropsychology, and continence. A score between 0 and 100 is determined for each domain (0 represents the most severe symptoms and 100 a complete absence of symptoms) and a total score between 0 and 100 is determined by averaging the score of the available domains (gait is weighted double). A subset of patients in the present study (*n* = 98) was evaluated with a version of the Stroop test (one of the neuropsychological tests) with only 24 boxes and words instead of 100, as in the original iNPH scale. The cognitive domain for these patients was determined using a conversion table based on normative data from a large set of iNPH patients, as described previously [33].

The difference in total iNPH scale score at the 12-month follow-up and at baseline was used to determine shunt outcome and is referred to below as the “delta total iNPH score”. Patients with delta total iNPH scores of ≥ 5 were defined as shunt responders [34]. Clinical evaluations were carried out by a specialized team of neurologists, neurosurgeons, nurses, physiotherapists, and occupational therapists. Gait velocity was assessed in a small subgroup (*n* = 11) of the cohort at the first evaluation, and again the day before shunt surgery to investigate if symptoms deteriorated while waiting for surgery. Levels of total tau (T-tau), phosphorylated tau (P-tau), amyloid beta1-42 (Aβ1-42), and neurofilament light chain protein (NfL) were analyzed from preoperative CSF samples, collected at the time of the baseline clinical evaluation. The methods for CSF sampling and biomarker analysis were previously described [33].

The following comorbidities were recorded during the preoperative workup: diabetes mellitus, hyperlipidemia, hypertension, history of acute myocardial infarction, history of ischemic stroke, aspirin use, and oral anticoagulant use. Comorbidities, among other baseline characteristics, are reported in Table 1.

**Table 1:** Demographic data, preoperative symptoms, and protein concentrations

Characteristic	Value	*n*
Age, years, mean (range)	75 (50–89)	253
Male, *n* (%)	141 (56%)	253
Comorbidity, *n* (%):		
Diabetes	63 (25%)	253
Hyperlipidemia	96 (38%)	253
Hypertension	162 (64%)	253
Previous AMI	37 (15%)	253
Previous stroke	32 (13%)	253
Aspirin use, *n* (%)	102 (40%)	253
Oral anticoagulant drug use*, *n* (%)	18 (7%)	253
Total iNPH scale score, mean (SD)	49.8 (19.0)	253
Separate domain scores, mean (SD)		
Balance domain	64.3 (20.9)	244
Continence domain	59.5 (26.1)	246
Gait domain	38.9 (24.1)	253
Cognitive domain	48.9 (21.2)	98
MMSE score, median (IQR)	25.0 (22.0–28.0)	249
Time between preoperative evaluation and surgery, months, median (IQR)	6 (4–8)	253
Protein concentrations, ng/L, median (IQR)		
T-tau	211 (150–326)	240
P-tau	30 (23–39)	236
Aβ1-42	540 (378–708)	237
NfL	1200 (790–1730)	75

*AMI* acute myocardial infarction, *iNPH* idiopathic normal pressure hydrocephalus, *SD* standard deviation, *MMSE* mini-mental state examination, *IQR* interquartile range, *ng* nanogram, *T-tau* total tau, *P-tau* phosphorylated tau, *Aβ1-42* amyloid beta1-42, *NfL* neurofilament light chain protein

*Warfarin or new oral anticoagulants

### Imaging protocol

All patients were investigated with preoperative MRI of the brain. Since the examinations were initiated clinically during the workup, there was no strict conformity in scanners and imaging protocols. However, all scans included a FLAIR sequence, as well as routine morphological images for diagnostic purposes. Evans’ index and disproportionately enlarged subarachnoid space hydrocephalus (DESH), both commonly used variables in the setting of iNPH, were assessed on preoperative computed tomography (CT) scans of the brain, as previously described [11, 35]. The scans were performed median 1 day (IQR 1–3) before the shunt surgery. Both Evans’ index and DESH were included as covariates in the regression analyses.

### Volumetric analyses

Volumes of DWMH, PVH, and the lateral ventricles were quantified on preoperative FLAIR images using SmartPaint, a semi-automatic volumetric segmentation software [30]. SmartPaint enables interactive segmentation of medical images using a freehand painting tool. The software takes both spatial and range distance (i.e., the difference in intensity values between voxels) into account when performing segmentation, allowing the user to easily outline relevant regions and quickly define volumes of interest. Both the spatial and range distance can be modified by the operator at any time during the segmentation, to adjust for differences in image contrast and the size of the structures to be segmented. Images uploaded in SmartPaint are obtained in an axial, coronal, and sagittal plane, and any segmentation performed in one plane is updated globally and displayed to the operator instantly. SmartPaint operates in three dimensions by default, enabling segmentation in multiple slices simultaneously [30]. All segmentations were performed in the axial plane and in the cranial to caudal direction throughout the entire cerebrum. The lateral ventricles were identified visually and segmented using the “brush tool” function in SmartPaint [30]. The volume was determined by multiplying the number of segmented ventricular voxels by the volume of a single voxel.

**Fig. 1:**
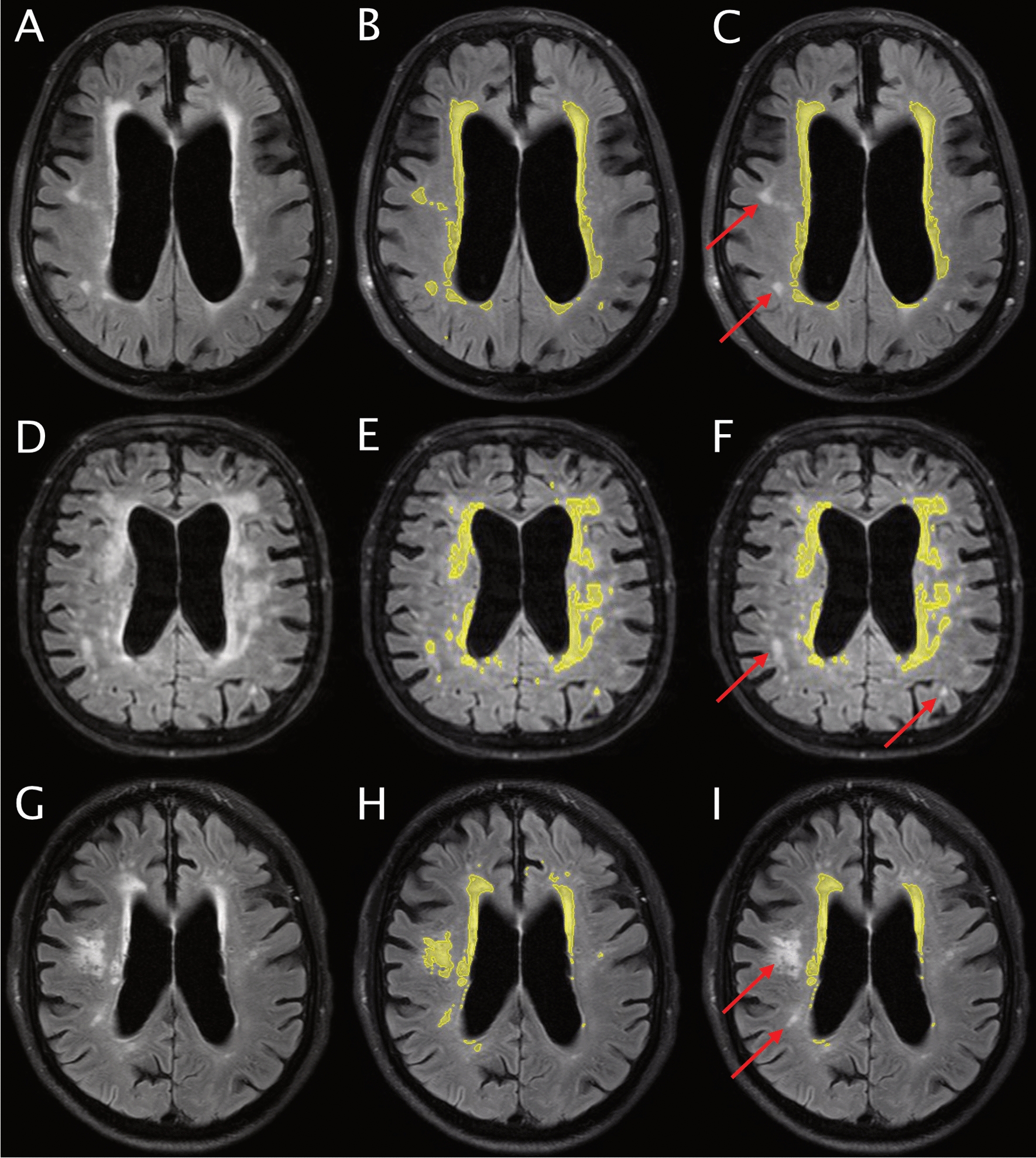
Brain images for three representative patients and the results from semi-quantitative segmentation of white matter changes. The left column (**A**, **D**, **G**) shows the original fluid-attenuated inversion recovery images and the middle column (**B**, **E**, **H**) shows the total white matter changes. The right column (**C**, **F**, **I**) shows the periventricular hyperintensities including adjacent changes, but excluding non-adjacent changes in deep white matter. The top row shows a patient with prominent periventricular hyperintensities and minor areas with changes in deep white matter. The middle row (**D**, **E**, **F**) shows a patient with extensive changes that are largely, but not exclusively, adjacent to the periventricular area. The bottom row shows a patient with modest changes in the periventricular area, but a large distinct area in deep white matter. The red arrows mark some of the areas detected as isolated deep white matter changes

WMC were defined as hyperintense lesions in white matter on FLAIR images [12] (Fig. 1) and segmented using a thresholding segmentation procedure, implemented in the SmartPaint software for this study. Maximum and minimum intensity values were set based on this function, and voxels with an intensity value within this range were automatically outlined. The maximum value was set at 100% in all subjects. The minimum value was customized for each subject, to ensure that all WMC were included. Other structures (e.g., hyperintense grey matter) with similar intensity as WMC were occasionally segmented in this procedure. These structures were identified and removed with the editing feature of the “brush tool” function [30]. WMC identified as “pencil-thin lining” adjacent to the ventricles have been reported as normal findings in healthy elderly people [36]. These were removed in all patients.

**Fig. 2:**
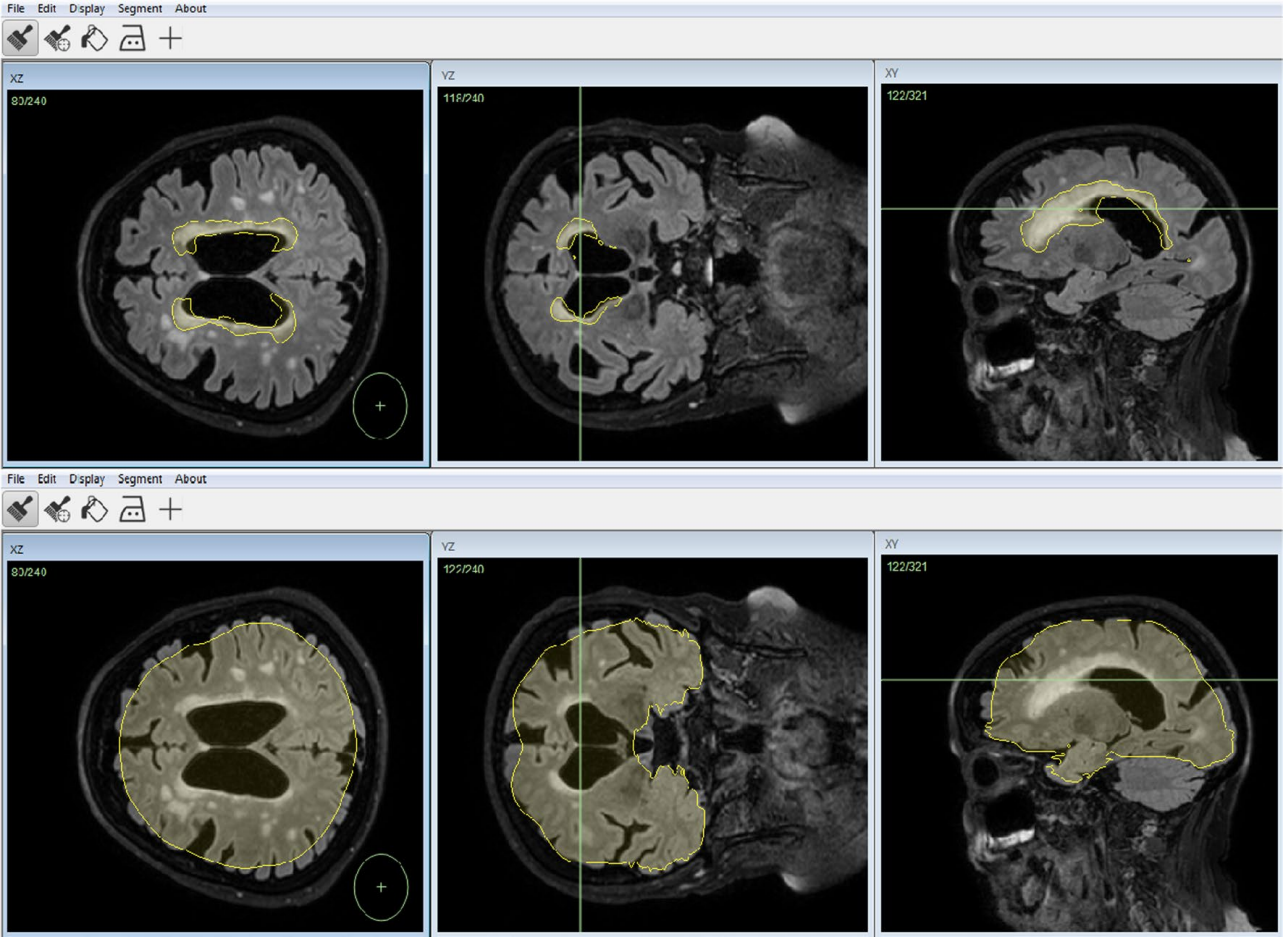
Two screenshots from the SmartPaint user interface illustrating the outlining of the two brain masks. The top row illustrates the outlining adjacent to the lateral ventricles (PVH mask) and the bottom row the outlining of the entire cerebrum (whole brain mask). The PVH mask was adjusted for each subject, depending on the width of PVH. DWMH were calculated by subtracting volume of WMC within the PVH mask from the total volume of WMC within the whole brain mask. Segmentations are shown as yellow semi-transparent overlays

Two brain masks were segmented with the “brush tool” function, to quantify WMC as either DWMH (separated from the lateral ventricles by normal-appearing white matter) or PVH (adjacent to the lateral ventricles). The first brain mask included voxels where both DWMH and PVH could exist (i.e., the entire cerebrum, equivalent to a whole brain mask), and the second brain mask included voxels where only PVH could exist (i.e., adjacent to the lateral ventricles, i.e., a PVH mask) (Fig. 2). The latter was adjusted for each subject, depending on the width of PVH. Since the volumetric analyses were performed only within these brain masks, hyperintense artifacts and structures outside the brain parenchyma (e.g., intraorbital structures) were excluded. The total volume of WMC and the volume of PVH were determined by multiplying the number of WMC voxels registered in each brain mask by the volume of a single voxel. The volume of DWMH was calculated by removing the volume of PVH from the total volume of WMC.

### SmartPaint validation

**Fig. 3:**
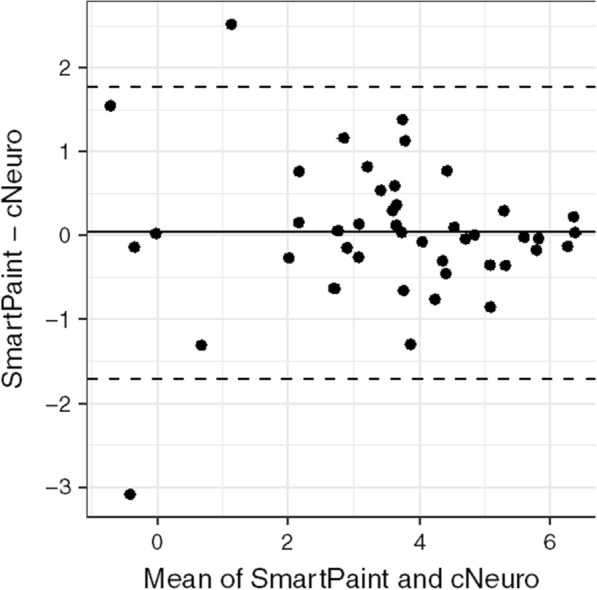
Bland–Altman plot illustrating agreement analysis between SmartPaint and cNeuro

In 44 patients, the total volume of WMC was measured in FLAIR sequences using cNeuro, a fully automated commercial segmentation tool (Combinostics Ltd, Tampere, Finland, https://www.combinostics.com/cmri/) [37]. Additionally, in 15 patients, the volume of the lateral ventricles was measured with SyntheticMR [38]. Intraclass correlation coefficients were determined by comparing the volumes generated with SmartPaint with the results of cNeuro (Fig. 3) and SyntheticMR, and were calculated to 0.895 (CI 95%, 0.817 to 0.941) and 0.939 (CI 95%, 0.736 to 0.982), respectively. The investigator was blinded to symptom and outcome scores.

### Statistics

The radiological volumes and biomarker values (T-tau, P-tau, Aβ1-42, and NfL) were log_2_-transformed in all statistical analyses. Continuous data were summarized as mean (SD) or median (IQR). The difference in symptom scores before and after surgery was assessed using the paired sample t-test (iNPH scale score) or Wilcoxon’s signed-rank test (MMSE score and domains). MRI variables were compared between shunt responders and non-responders using the Mann–Whitney’s U test. The correlation between PVH and DWMH was assessed using Spearman’s rank correlation coefficient. Associations between delta scores and radiological volumes were assessed using linear regression with delta scores as dependent variables and volumes, age, gender, time between preoperative evaluation and surgery, preoperative total iNPH scale score, Evans’ index, and DESH as independent variables. Associations between baseline symptom scores and radiological volumes were assessed using linear regression with baseline symptom scores as dependent variables and volumes, age, gender, Evans’ index, and DESH as independent variables. Associations between CSF biomarkers and radiological volumes were assessed using linear regression with CSF biomarkers as dependent variables and volumes, age, gender, Evans’ index, and DESH as independent variables. In all analyses, the significance level was set to 0.05 and no correction for multiple analyses was performed due to pre-defined statistical analyses.

## Results

Twelve months after surgery, the iNPH scale score was improved in 152 (60%) of the 253 patients, unchanged in 5 (2%), and reduced in 96 (38%). One hundred and twenty-nine (51%) patients had improved by ≥ 5 points on the iNPH scale and were defined as shunt responders. The largest improvement seen in a patient was 59 points. The MMSE score was improved in 110 (46%) patients, unchanged in 44 (18%), and reduced in 87 (36%). No significant difference in any of the radiological volumes was detected between the shunt responder group and the non-responder group (Table 2). In 11 patients investigated both at first evaluation and the day before shunt surgery (7 months (IQR 6–9)), gait velocity deteriorated from 0.71 m/s (IQR 0.43–0.83) to 0.63 m/s (IQR 0.36–0.77), p < 0.05.

**Table 2:** Radiological volumes in shunt responders and non-responders

Volume (ml)	Responders (*n* = 129)	Non-responders (*n* = 124)	P-value
PVH, median (IQR)	14.7 (5.8–28.7)	16.5 (6.3–36.6)	ns^*^
DWMH, median (IQR)	1.7 (0.7–3.3)	2.0 (0.8–4.3)	ns^*^
Lateral ventricles, mean (SD)	129.7 (42.3)	128.2 (41.1)	ns^*^

*ml* milliliter, *PVH* periventricular hyperintensities, *IQR* interquartile range, *ns* non-significant, *DWMH* deep white matter hyperintensities, *SD* standard deviation

*Mann–Whitney U-test

The effect of radiological volumes on outcome after shunt surgery (delta scores) was investigated through linear regressions (Table 3). A larger volume of PVH was negatively associated with shunt outcome (delta total iNPH score) when adjusting for age, gender, waiting time for surgery, preoperative total iNPH scale score, Evans’ index, and DESH, *B* = − 1.173, p = 0.0309. When investigating the outcome of separate symptom domains, a larger volume of PVH was associated with less improvement in gait symptoms, *B* = − 2.068, p = 0.00314. The volume of DWMH was not associated with shunt outcome (p = 0.0896). No radiological volume was significantly associated with any baseline preoperative symptom score when adjusting for age, gender, Evans’ index, and DESH (Table 4). There was a strong positive correlation between the volumes of PVH and DWMH, r_s_ = 0.752 p < 0.001.

**Table 3:** Linear regression with shunt surgery outcome (delta scores) as dependent variables and radiological volumes as independent variables

Delta score	Volume	Unadjusted		Adjusted		*n*
		*B*	*P-*value	*B*	*P-*value	
Delta total iNPH score	PVH	− 1.260	0.0269*	− 1.173	0.0309*	253
	DWMH	− 0.816	0.0899	− 0.761	0.0896	253
	Lateral ventricles	1.752	0.501	4.002	0.229	253
Delta balance score	PVH	− 0.557	0.357	− 0.365	0.518	238
	DWMH	− 0.481	0.35	− 0.086	0.856	238
	Lateral ventricles	0.873	0.756	3.911	0.259	238
Delta continence score	PVH	0.048	0.959	− 0.020	0.982	235
	DWMH	− 0.241	0.755	− 0.183	0.809	235
	Lateral ventricles	4.600	0.282	13.337	0.0175*	235
Delta gait score	PVH	− 2.148	0.00352**	− 2.068	0.00314**	252
	DWMH	− 1.071	0.0861	− 1.073	0.0636	252
	Lateral ventricles	1.172	0.729	0.157	0.971	252
Delta cognitive score	PVH	− 0.042	0.95	0.047	0.944	95
	DWMH	− 0.632	0.263	− 0.659	0.231	95
	Lateral ventricles	0.564	0.86	0.690	0.875	95
Delta MMSE score	PVH	− 0.201	0.0703	− 0.146	0.196	241
	DWMH	− 0.128	0.167	− 0.059	0.519	241
	Lateral ventricles	1.242	0.0138*	1.283	0.063	241

Data are adjusted for age, gender, time between preoperative evaluation and surgery, preoperative total iNPH scale score, Evans’ index, and DESH

*iNPH* idiopathic normal pressure hydrocephalus, *DESH* disproportionately enlarged subarachnoid space hydrocephalus, *B* unstandardized regression coefficient, *PVH* periventricular hyperintensities, *DWMH* deep white matter hyperintensities, *MMSE* mini-mental state examination. Bold values are significant, * < 0.05, ** < 0.01

**Table 4:** Linear regression with baseline symptom scores as dependent variables and radiological volumes as independent variables

Baseline score	Volume	*B*	*P-*value	*n*
Total iNPH scale	PVH	− 0.688	0.242	253
	DWMH	0.075	0.878	253
	Lateral ventricles	3.481	0.333	253
Balance domain	PVH	0.367	0.586	244
	DWMH	1.046	0.0575	244
	Lateral ventricles	3.185	0.44	244
Continence domain	PVH	− 0.303	0.717	246
	DWMH	0.236	0.732	246
	Lateral ventricles	3.862	0.452	246
Gait domain	PVH	− 1.234	0.0979	253
	DWMH	− 0.458	0.458	253
	Lateral ventricles	1.491	0.745	253
Cognitive domain	PVH	− 1.692	0.102	98
	DWMH	− 0.004	0.996	98
	Lateral ventricles	− 8.171	0.231	98
MMSE	PVH	− 0.093	0.489	249
	DWMH	0.170	0.127	249
	Lateral ventricles	− 1.221	0.142	249

Data are adjusted for age, gender, Evans’ index, and DESH

*DESH* disproportionately enlarged subarachnoid space hydrocephalus, *B* unstandardized regression coefficient, *iNPH* idiopathic normal pressure hydrocephalus, *PVH* periventricular hyperintensities, *DWMH* deep white matter hyperintensities, *MMSE* mini-mental state examination

Both the volume of DWMH (p < 0.01) and the volume of PVH (p < 0.05) were larger in patients with hypertension than in patients without hypertension. There was no difference in volumes in the presence or absence of diabetes mellitus or hyperlipidemia.

Levels of NfL, T-tau, P-tau, and Aβ1-42 were analyzed from preoperative CSF samples. The median concentrations of the biomarkers are reported in Table 1. Neither the volume of DWMH nor the volume of PVH was associated with any preoperative biomarker when adjusted for age, gender, Evans’ index, and DESH. A larger volume of the lateral ventricles was associated with lower levels of T-tau, *B* = − 0.474, p = 0.00701 (Table 5).

**Table 5:** Linear regression with CSF biomarkers as dependent variables and radiological volumes as independent variables

Biomarker	Volume	*B*	*P-*value	*n*
T-tau	PVH	0.008	0.772	240
	DWMH	0.009	0.717	240
	Lateral ventricles	− 0.474	0.00701**	240
P-tau	PVH	− 0.011	0.58	236
	DWMH	− 0.019	0.273	236
	Lateral ventricles	− 0.135	0.287	236
Aβ1-42	PVH	− 0.032	0.091	237
	DWMH	− 0.017	0.264	237
	Lateral ventricles	− 0.185	0.11	237
NfL	PVH	0.082	0.0852	75
	DWMH	0.048	0.173	75
	Lateral ventricles	− 0.103	0.74	75

Data are adjusted for age, gender, Evans’ index, and DESH

*CSF* cerebrospinal fluid, *DESH* disproportionately enlarged subarachnoid space hydrocephalus, *B* unstandardized regression coefficient, *T-tau* total tau, *PVH* periventricular hyperintensities, *DWMH* deep white matter hyperintensities, *P-tau* phosphorylated tau, *Aβ1-42* amyloid beta1-42, *NfL* neurofilament light chain protein. Bold values are significant, * < 0.05, ** < 0.01

## Discussion

The main finding of this study was that there was no significant difference in preoperative volume of WMC between shunt responders and non-responders, when shunt response was defined as improvement with ≥ 5 points on the total iNPH scale 12 months after surgery. However, a larger volume of PVH was associated with a less favorable response to surgery. The volume of DWMH was not associated with shunt outcome. This is, to our knowledge, the largest (*N* = 253) study assessing the predictive value of WMC in iNPH patients using a volumetric method.

Many studies that have graded the extent of WMC in patients with NPH have used non-volumetric, subjective rating scales [9, 24, 25, 29]. Most studies that have investigated the predictive value of WMC have combined the results of patients with iNPH and those with secondary NPH [9, 25–28], using the collective term NPH. In this study, a semi-automatic volumetric method was used to quantify DWMH and PVH separately, and only in patients diagnosed with iNPH.

### Outcome after shunt surgery

Previous studies have shown that 60–80% of patients with iNPH have improved 12 months after shunt surgery, measured on the iNPH scale [7, 8]. The low proportion of shunt responders (51%) in this study is probably a result of the long waiting times for surgery (median 6 months). Long waiting times negatively affect shunt outcome [23] and iNPH patients deteriorate while waiting for surgery [39]. Since a subgroup of our cohort deteriorated in gait velocity with 0.08 m/s between the baseline evaluation and the day before shunt surgery, it is plausible to assume that more patients probably had worse symptoms at the time of surgery compared with at the clinical evaluation 4–8 months earlier, resulting in falsely high baseline symptom scores (i.e., an underestimation of severity). Based on this, we recommend that if a significant amount of time has passed between preoperative evaluation of symptoms and shunt surgery, then the patient’s iNPH exam should be performed again, which will help to document whether, on an individual basis, worsening has occurred, leading to a more correct assessment of outcome.

### Origin and pathophysiology of white matter changes

iNPH is associated with the presence of WMC on preoperative brain imaging [10], in addition to hydrocephalic features. Reduction in ventricular size after shunt surgery in iNPH patients does not seem to correlate with clinical improvement [9, 40], and pathophysiological mechanisms beyond mechanical compression of the brain parenchyma are likely involved.

DWMH have been associated with vascular comorbidities such as hypertension and lacunar infarcts [41–43], and it is often assumed that these peripheral lesions represent ischemic tissue damage, secondary to cerebral small vessel disease [9, 14, 25, 44]. Postmortem studies have reported increased hypoxia-related factors in DWMH [45]. Punctate lesions may also be related to widened perivascular spaces (Virchow-Robin spaces) [46].

Smooth PVH (caps and halo phenomena) have been associated with subependymal gliosis and disruption of the ependymal lining [12, 14, 47], and the severity of PVH has been associated with loss of ventricular ependyma in postmortem studies [45]. These structural alterations may predispose for leakage of ventricular CSF into adjacent brain parenchyma [12, 14], causing extracellular edema. Furthermore, the periventricular area is prone to focal and systemic hypoperfusion due to watershed blood supply [12, 48, 49]. Irregular PVH have been associated with large vessel disease such as aortic and carotid atherosclerosis [13, 50] and may be the result of chronic hemodynamic insufficiency with subsequent ischemic demyelination and loss of axons [12, 47, 48, 51].

In line with previous work [10], a strong correlation between DWMH and PVH was observed in the present study. Co-occurrence may be partly explained by the high prevalence of vascular risk factors in this patient group, and hypertension was associated with both PVH and DWMH in this study sample. However, an important contributing factor is that it can be difficult or even impossible to distinguish between these lesions in some cases.

### Clinical significance of white matter changes

DWMH and PVH on preoperative brain imaging have been associated with more severe symptoms in iNPH patients [9, 25, 29]. PVH tend to decrease after shunting, and this phenomenon is associated with symptomatic relief [9, 25, 52]. Somewhat unexpectedly, we observed that a larger volume of WMC was not associated with worse symptoms at baseline, indicating that other mechanisms were more important for the symptom burden.

Previous studies have reported that periventricular hypodensities on CT scanning predict a favorable response to shunt surgery in NPH patients [26, 27]. In more recent studies, the presence of DWMH or PVH on preoperative MRI has been associated with unfavorable shunt outcome in iNPH patients [29], but it is reported that patients with widespread WMC may still benefit from shunt surgery [29, 53]. Other studies have reported that WMC lack prognostic value [9, 24] and that the extent of WMC does not differ between shunt responders and non-responders [9]. The presence of DWMH or PVH have also been associated with favorable shunt outcome in NPH patients [25].

In the present study, we report that no MRI variable differed between shunt responders and non-responders, indicating that iNPH patients with WMC may benefit from shunt surgery. Even though a larger volume of PVH was associated with less favorable shunt outcome, our results indicate that the presence of such lesions should generally not exclude patients from receiving shunt surgery.

Elevated NfL concentrations in preoperative lumbar CSF have been associated with WMC in iNPH patients [25]. In a large sample study, Braun et al. recently reported that NfL in CSF was associated with worse outcome in iNPH patients, which could be attributed to more damage in white matter structures [33]. Decreasing levels of NfL following surgery are associated with clinical improvement [54], and the same phenomena may be the case for WMC. However, there was no significant association between NfL and WMC in this study. Lower levels of T-tau were associated with a larger ventricular volume, which was probably due to a dilution effect from enlarged CSF spaces.

### Limitations

The long waiting times for surgery in the present study probably resulted in a low proportion of shunt responders, as described above. However, waiting time for shunt surgery was adjusted for in the regression analyses (Table 3) so this limitation probably did not affect the main results. Even with careful postoperative evaluations there is a risk of missed shunt failures that could negatively affect the proportion of shunt responders. The time from onset of symptoms has been described as a predictor of shunt outcome [55, 56], but was not included in statistical analyses in this study, mainly due to the poor reliability of this variable.

The sensitivity for WMC can differ slightly between FLAIR sequences on different scanners, but this difference was considered small in comparison to the large volumes of WMC encountered in most patients. Distinguishing between DWMH and PVH can be difficult and has been addressed and discussed in several papers. The issue is recognized as controversial and no method known to us offer a simple solution. In one study, it was impossible to distinguish between DWMH and PVH in one-third of patients [9]. In patients with advanced WMC, DWMH and PVH often tend to coalesce and the continuity-to-ventricle rule may not be applicable. Defining DWMH and PVH based on distance [57] would allow for improved consistency and method reproducibility. However, this definition is problematic from a physiological and pathological perspective [12]. A head-to-head comparison between different classification methods found that the continuity-to-ventricle rule and the 10 mm-rule yielded highly similar results and the authors concluded that the exact method should not be considered a major obstacle [58]. Only a subgroup of the cohort (39%) was investigated with the cognitive domain of the iNPH scale, and therefore the MMSE was also included as a cognitive test. The MMSE underestimates subcortical deficits and is susceptible to practice effects and is therefore not an optimal test in patients with iNPH.

Comorbidity was reported as categorical variables in this study and many other studies in this field. In future research, it may facilitate comparisons between studies and improve statistical analyses if comorbidity was instead reported as continuous variables, preferably as a combined scale or index. There is always a risk of inclusion bias in retrospective studies such as this one, as we only included patients that were selected for shunt surgery. The MRI used in this study were assessed in the preoperative work-up and it is possible that the extent of WMC in selected cases influenced the decision to not recommend shunt surgery.

Like other interactive segmentation tools, SmartPaint involves a trade-off between the time spent on segmentation and the accuracy of the results [30]. The mean time spent on producing a visually satisfying segmentation was 25 min per subject.

## Conclusions

An association between outcome after shunt surgery and the volume of PVH was seen, but there was no difference between shunt responders and non-responders regarding the volumes of PVH or DWMH. We conclude that preoperative assessment of WMC should not be used to exclude patients with iNPH from shunt surgery. SmartPaint is a promising interactive tool, limiting time spent on manual segmentation while maintaining high result accuracy.

## Data Availability

Anonymized datasets analyzed during the current study are available from the corresponding author on reasonable request.

## Abbreviations

iNPH: Idiopathic normal pressure hydrocephalus
CSF: Cerebrospinal fluid
WMC: White matter changes
DWMH: Deep white matter hyperintensities
PVH: Periventricular hyperintensities
MRI: Magnetic resonance imaging
FLAIR: Fluid-attenuated inversion recovery
NfL: Neurofilament light chain protein
T-tau: Total tau
P-tau: Phosphorylated tau
Aβ1-42: Amyloid beta1-42
IQR: Interquartile range
MMSE: Mini Mental State Examination
